# *Salmonella* as a Model for Non-Cognate Th1 Cell Stimulation

**DOI:** 10.3389/fimmu.2014.00621

**Published:** 2014-12-10

**Authors:** Hope O’Donnell, Stephen J. McSorley

**Affiliations:** ^1^Department of Anatomy, Physiology and Cell Biology, Center for Comparative Medicine, School of Veterinary Medicine, University of California Davis, Davis, CA, USA; ^2^Microbiology, Immunology, and Cancer Biology Graduate Program, University of Minnesota Medical School – Twin Cities, Minneapolis, MN, USA

**Keywords:** *Salmonella*, CD4 T cell, Th1 cell, innate stimulation, TLR, NLR, IFN-γ

## Abstract

*Salmonella* has been a model pathogen for examining CD4 T cell activation and effector functions for many years due to the strength of the Th1 cell response observed during *Salmonella* infections, the relative ease of use of *Salmonella*, the availability of *Salmonella*-specific T cell reagents, and the well-characterized nature of the model system, the pathogen, and the immune response elicited. Herein, we discuss the use of *Salmonella* as a model pathogen to explore the complex interaction of T cells with their inflammatory environment. In particular, we address the issue of bystander activation of naïve T cells and non-cognate stimulation of activated and memory T cells. Further, we compare and contrast our current knowledge of these non-cognate responses in CD8 versus CD4 T cells. Finally, we make a case for *Salmonella* as a particularly appropriate model pathogen in the study of non-cognate CD4 T cell responses based on the strength of the Th1 response during infection, the requirement for CD4 T cells in bacterial clearance, and the well-characterized inflammatory response to conserved molecular patterns induced by *Salmonella* infection.

## Introduction

T cell activation and effector functions have been extensively studied *in vitro*, allowing for controlled interactions within a defined environment. However, studying T cells *in vitro* inherently limits interactions to those that have been previously defined. To explore more complex systems of interactions beyond known parameters requires using an *in vivo* model system. One common technique for studying T cell responses *in vivo* is to examine a population of T cells with known antigen specificity. This includes the use of T cell receptor (TCR) transgenic mice, model antigens like ovalbumin, and major histocompatibility complex (MHC) class I and II tetramers presenting defined peptide sequences, which allows for the detection of T cells specifically recognizing that peptide. These reagents have greatly facilitated the tracking of antigen-specific T cells and the study of monoclonal T cell responses. Together with *in vitro* studies, the examination of antigen-specific T cells *in vivo* has been essential in defining much of what we know about T cell immunology.

When trying to understand the diverse polyclonal responses that are induced by infections, *in vivo* techniques that examine individual antigen-specific responses are likely to be limited. The natural breadth of the naïve TCR repertoire is an important strength of the adaptive immune response and can only be maintained by having pools of individual clones at very low frequency. Recent evidence has shown that altering the frequency of a given T cell clone can impact the activation strength, kinetics, and memory formation of the resulting T cell response (1–4). This issue complicates TCR transgenic mouse studies, which focus on a monoclonal population, generally used at unnaturally high frequency. Studying the natural endogenous precursor population is therefore important and also complex since the frequency of individual clones also varies within the naïve repertoire (5).

Furthermore, individual TCR specificities may be predisposed toward different fates (6) and may also be regulated by temporal and anatomical antigen expression by the pathogen, factors that might significantly affect some clonal populations differently than the overall polyclonal T cell response (7, 8). These issues affect the use of TCR transgenic mice, MHC tetramer studies, and model antigens, because it may lead to a situation where the T cell response under study may not be representative of the overall T cell response to the pathogen. Likewise, studies that attempt to activate T cells with model antigens in the absence of infection are unlikely to accurately reflect the complex interactions that occur between T cells and the rest of the immune system in the context of a strong inflammatory response. Thus, to examine the full range of T cell functions and interactions within the larger immune network, it is necessary to study them in the context of a natural polyclonal response that includes a broad range of antigens and the inflammatory milieu that differentiates infection from other surrogate means of activation.

When exploring the responses of CD4 T cells, in particular, it is critical to examine their functions under circumstances in which they are naturally induced and required. In other words, it makes very little sense to study the effector function of Th1 cells using models where these Th1 cells do not contribute to pathogen clearance. The role of the Th1 subset of CD4 T cells and its effector cytokine IFN-γ in *Salmonella* infections has been very well established (9–11), making *Salmonella* model systems particularly appropriate for characterizing Th1 cell functions. Additionally, the innate immune response and inflammatory responses occurring during *Salmonella* infections are relatively well-defined (12–16), making it an ideal model to characterize the influence of natural inflammatory conditions on these Th1 cell responses.

In this review, we will highlight the unique advantage of the *Salmonella* model system for studying Th1 responses to innate stimuli. First, in Section “Armed and Ready: T Cell Responses to Innate Signals,” we discuss and compare conventional cognate T cell stimulation, non-cognate stimulation of activated conventional T cells, and the responses of innate-like T cells. Thus far, most studies examining non-cognate T cell responses have focused on CD8 T cells, primarily in viral infection models. It is likely that the rules governing non-cognate CD8 T cell responses differ in certain aspects to those governing non-cognate responses in CD4 T cells. However, comparing these responses in infection models that generate overall weak CD4 T cell responses due to poor activation does not allow accurate comparison of the capacity of the non-cognate CD4 T cell response. In Section “A Complicated Relationship: The Dynamic Interactions of *Salmonella* and Th1 Cells,” we discuss the dynamic interaction of *Salmonella* and the Th1 response, focusing on the important role of Th1 cells in the resolution of *Salmonella* infection, and potential ways that *Salmonella* might be able to subvert cognate T cell recognition and thus increase the requirement for non-cognate recognition pathways.

## Armed and Ready: T Cell Responses to Innate Signals

### Conventional T cell responses

**Figure 1:**
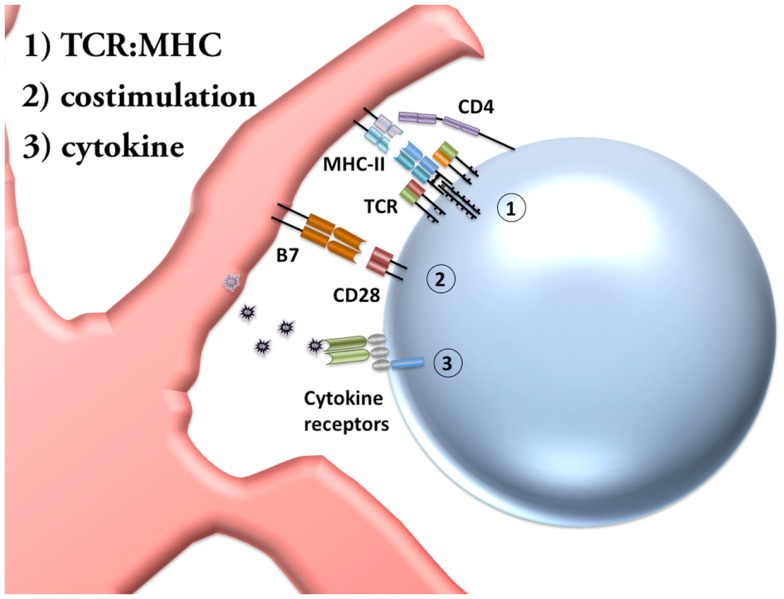
**Priming of CD4 T cells requires three signals**. Conventional activation of naïve CD4 T cells requires three distinct signals. Signal 1: the TCR (T cell receptor) on the T cell must recognize a particular peptide sequence, processed within an APC (antigen presenting cell) and presented by MHC-II (major histocompatibility complex-II) in mice. The CD4 co-receptor shown stabilizes the T cell interaction with MHC-II. Signal 2: activated DCs (dendritic cells) upregulate the costimulatory molecules B7.1 and B7.2 (also called CD80 and CD86). CD28 on T cells recognize these costimulatory molecules as a second signal for activation. In the absence of costimulation T cells undergo anergy or death. Signal 3: the cytokine environment instructs the final stage of T cell priming by determining the differentiation pathway undergone by the activated T cell.

Before discussing innate stimulation of T cells, we will first briefly review initial T cell activation, differentiation, secondary stimulation at sites of infection, and formation of memory. Both CD4 and CD8 T cells are activated upon recognition of a specific peptide sequence in host MHC by the TCR. CD8 T cells recognize this peptide presented within MHC-I expressed on most cell surfaces, while CD4 T cells interact with antigen presented in MHC-II only by antigen presenting cells (APCs). These APCs, often dendritic cells (DCs), collect and process antigen in the periphery and return to the lymph nodes and other secondary lymphoid organs with the antigens presented on their surface, where they can be recognized by interacting T cells. This first antigen-specific interaction is referred to as signal 1 of T cell activation (Figure 1) because, although it is required, the TCR stimulation alone is not sufficient to functionally activate the T cell. On its own this TCR interaction will ultimately lead to anergy, a state of unresponsiveness that maintains peripheral tolerance.

During the initial TCR:MHC interaction, the T cell requires a second signal for the priming process. This second signal is referred to as costimulation (Figure 1), and can be achieved by a number of different interactions, although the most common activating signal is between CD28 on CD4 T cells and B7 molecules on DCs. The expression of these costimulatory molecules are upregulated on DCs after DC activation by inflammation, increasing the likelihood that T cells will be activated only when the antigen is encountered by the DC under appropriate conditions. While this second signal prevents anergy, a third signal is required to complete CD4 T cell priming and instruct the differentiation pathway.

Differentiation is a critical step in CD4 T cell priming because of the eclectic capacities of CD4 T cells. Once a CD4 T cell has recognized antigen presented by MHC-II and a costimulation signal, a third cytokine signal (Figure 1) will instruct the CD4 T cell to differentiate into a subset trained for a particular function. In this review, we focus on the Th1 subset of CD4 T cells, in which interleukin-12 (IL-12) allows sustained upregulation of the transcription factor T-bet and, upon re-stimulation, production of effector cytokines such as IFN-γ, TNFα, and IL-2. Additional CD4 subsets include Th2 (which respond to IL-4 and are regulated by GATA3), Th17 (combinations of TGF-β, IL-6, IL-21, and IL-23 lead to RORγt expression), Tfh (T follicular helper, respond to IL-6 and IL-21 to upregulate Bcl-6), and iTreg cells (induced T regulatory, TGF-β and IL-2 lead to expression of Foxp3), as well as other, less well-characterized subsets of CD4 T cells. It is important to note, however, that substantial evidence now supports the impermanence of some of these differentiation pathways, a concept known as effector plasticity. Thus, while CD4 T cells require these initial differentiation instructions, they often retain the capacity to acquire new functions under sufficient alternative stimulation.

The initial process of T cell activation dramatically alters the cell, causing upregulation of cascades of transcription factors, as well as altering the miRNA regulation, epigenetic modifications, and post-translational pathways. These changes program the cell with the capacity to rapidly respond upon re-stimulation in a specialized manner. However, the actual secretion of effector cytokines still requires some regulation to prevent unnecessary inflammation. For this reason, activated T cells arrive at sites of infection primed for rapid response, but not constitutively secreting cytokines. Traditionally, the secondary interactions of activated T cells at sites of infection are believed to consist of additional antigen-dependent interactions of the TCR and peptide-MHC complexes, which triggers a transient robust effector response in the appropriate location (17). Unlike the initial activation process, antigen-specific interaction alone is sufficient to induce cytokine production, because of the T cell’s activated state (18). However, much of this work has been conducted in low-inflammatory conditions and focused on the requirement for TCR interactions. Such work does not rule out a role for inflammatory stimulation of T cells during infections. Indeed, the ability of inflammatory cytokines to either activate naïve T cells or stimulate cytokine secretion from effector T cells will be discussed in the next section.

After initial activation, T cells undergo massive clonal expansion. This expansion of specific effector Th1 cells typically takes a few days, and in prolonged infections like *Salmonella* this T cell response can take a few weeks to reach the peak of expansion. During this time, T cells are responding to a complex network of signals, including IL-7 to survive, IL-2 to proliferate, pro-inflammatory cytokines, anti-inflammatory cytokines, and potentially secondary TCR signals. The combination of these encounters does more than just stimulate T cells to produce effector cytokines – it establishes their fate. While T cell responses are critical to pathogen clearance in many cases, they also have the potential to damage host tissues and for this reason, most T cells will ultimately be instructed to die. Thus, after the peak of clonal expansion T cells undergo a contraction phase in which most T cells receive apoptotic signals and are removed from circulation.

However, some T cells receive just the right combination of stimuli and survival signals to transition from an “armed and ready” effector state to a quiescent memory state. CD4 T cells can exist in a number of different memory states, which may ultimately affect their longevity, the areas in which they circulate, and the requirements for re-activation. The best described examples of CD4 memory subsets are the central versus effector memory T cells, which circulate in lymphoid tissue or non-lymphoid tissue, respectively. CD8 T cells are also believed to form these subsets, as well as memory populations called short-lived effector cells (SLECs) or memory precursor effector cells (MPECs) whose formation depends heavily on the inflammatory signals received, but which have not been described for CD4 T cells. Memory T cells are an important component of the rapid response to re-challenge with previously encountered pathogens because of their lowered activation threshold, pre-differentiated state, and extensive epigenetic modifications that allow for rapid relay of the signals needed to elicit effector function. Understanding how these memory T cells are formed and are able to respond is especially crucial to vaccine design.

**Figure 2:**
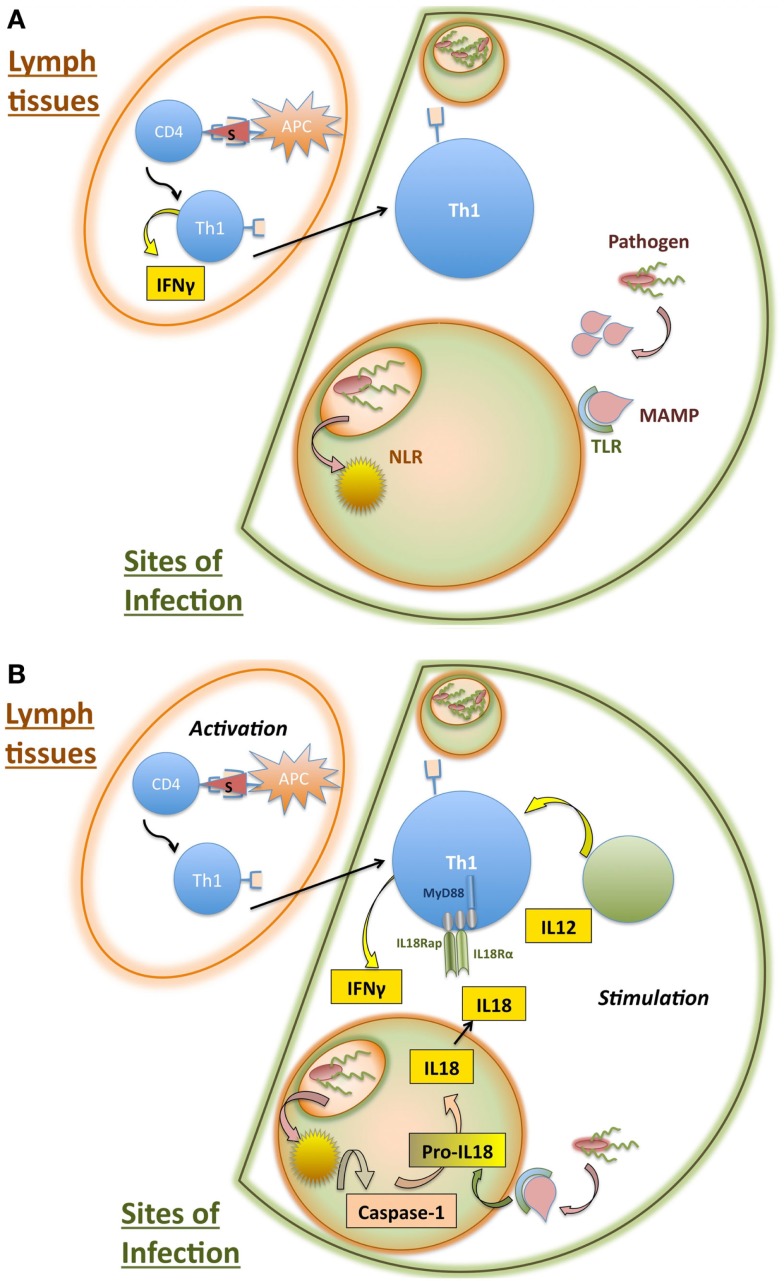
**Partial mechanism for non-cognate stimulation of activated CD4 T cells**. **(A)** Initiation of innate and adaptive immune responses during intracellular infections. CD4 T cells are initially activated in lymph tissues upon recognition of particular peptide antigens presented by antigen presenting cells (APCs). During intracellular infections, the presence of IFN-γ and IL-12 results in differentiation of these activated CD4 T cells into Th1 cells, which produce IFN-γ. The Th1 cells then traffic to sites of infection, where they require additional stimulation to induce production of effector cytokines. Meanwhile, sites of infection experience inflammation elicited by innate recognition of pathogens. Pattern recognition receptors such as toll-like receptors (TLRs) and nod-like receptors (NLRs) recognize conserved products of infection, called microbe-associated molecular patterns (MAMPs). **(B)** Innate inflammation stimulates Th1 cells to amplify effector response at sites of infection. TLR recognition of innate ligands results in the upregulation of pro-IL-18, while NLR recognition of infection activates caspase-1. Caspase-1 cleavage of pro-IL-18 into the mature form of the cytokine IL-18 allows secretion. IL-18 receptor is required by Th1 cells for non-cognate elicitation of IFN-γ. CD4 T cell stimulation at sites of infection likely involves additional cytokine pathways, including IL-12, which can act synergistically with IL-18 to stimulate Th1 cells.

**Figure 3:**
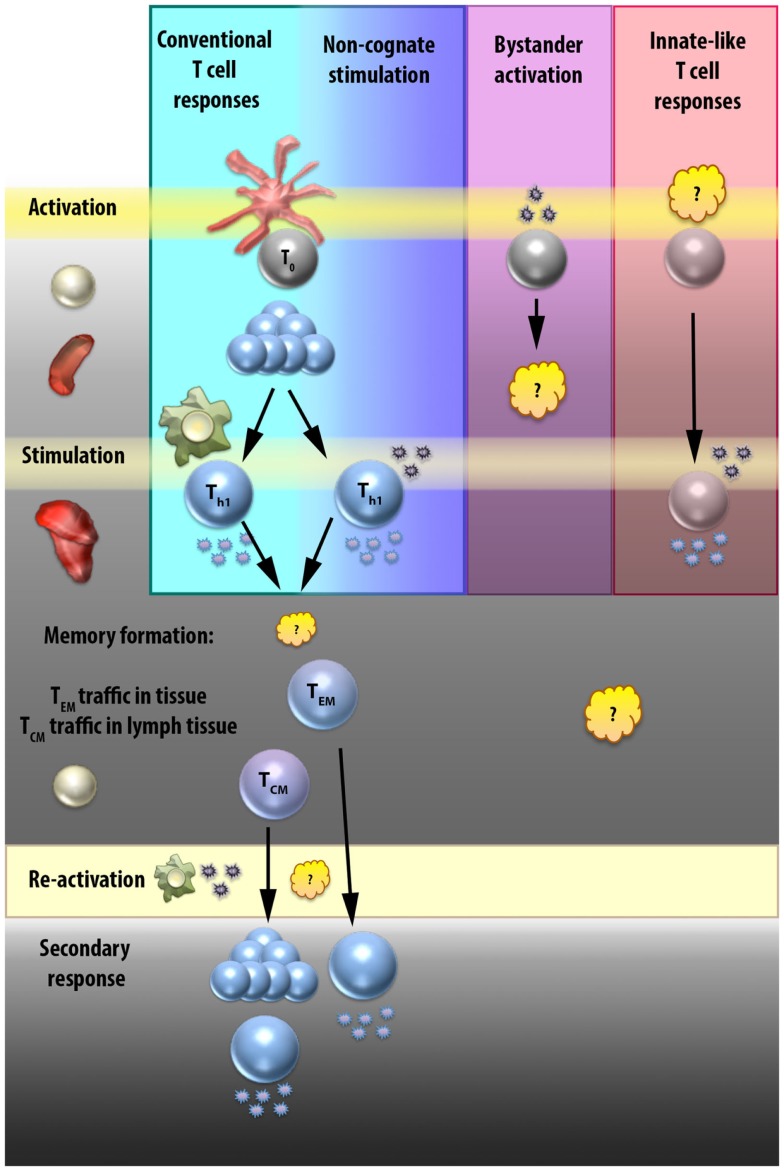
**Elicitation of CD4 and “innate-like” T cell effector functions**. T cell effector functions can result from interactions with APCs or cytokines at various stages. In the conventional T cell response (first column, blue), naïve T cells are activated by direct interactions with APCs in the secondary lymphoid tissues, proliferate, migrate to sites of infection, and then are stimulated by secondary direct APC contact to produce effector cytokines. Some of these effector T cells will go on to become memory T cells. T central memory (T_CM_) cells circulate in secondary lymphoid tissues, and upon re-activation will once again proliferate and differentiate into effector cells. T effector memory (T_EM_) cells can either migrate in the periphery or be resident in tissue, and respond more rapidly than the T_CM_ cells because upon re-activation they can secrete effector cytokines directly. The signals that result in the T_EM_ or T_CM_ fate decision are still unclear, as are the interactions required by each for re-activation, although it is presumed that re-activation occurs after direct interaction with an APC. Non-cognate stimulation (second column, indigo) occurs at sites of infection in T cells that have already undergone conventional activation in the secondary lymphoid tissues. Instead of being stimulated by secondary direct APC contact, these cells receive stimulatory signals from cytokines that induce IFN-γ production. Whether these cells go on to join the memory pool and how this different stimulation signal affects the fate decision of CD4 T cells is unknown. Bystander activation is a term that has been used loosely to mean any TCR-independent T cell stimulatory interaction. In the third column (purple), we focus on the idea of bystander activation as a mechanism to prime a CD4 T cell in a TCR-independent manner. While the effect of cytokines on naïve T cells have been studied at length *in vitro*, there is limited evidence that a naïve CD4 T cell can be activated by cytokine signals alone, and no evidence that TCR-independent activation can produce a fully functional effector T cell. The last column (pink) provides a general representation of innate-like T cell subsets. Although the initial priming signals for the different innate-like T cell populations vary and are still unclear in some cases, they include alternative activation mechanisms such as restricted TCRs or constitutive priming. Stimulation of effector responses at sites of infection in innate-like T cells is known to occur rapidly in response to cytokine stimulation, hence the name “innate-like” T cells. However, it is possible that all T cells have the capacity, once activated, to respond rapidly to cytokine stimulation, and that what really separates these innate-like cell types are their unique priming mechanisms.

While the above mechanisms comprise a very basic understanding of conventional T cell activation, there are a number of caveats and exceptions that are worth discussing. In Section “Bystander Activation and Non-Cognate Stimulation,” we will discuss the ability of T cells to respond to non-TCR stimuli, focusing on what has been referred to in the literature alternatively as bystander activation or non-cognate stimulation. A partial mechanism for non-cognate stimulation of Th1 cells is illustrated in Figure 2. In Section “Innate-Like T Cells and ILCs,” we will outline some of the non-conventional T cell subsets that are able to respond to non-cognate stimuli to draw parallels between these “innate-like” cell types and the innate-like functions of classically activated T cells. Figure 3 compares the interactions that occur in each of these cell responses and highlights areas that require further elaboration.

### Bystander activation and non-cognate stimulation

The strict processes governing conventional T cell activation are important to avoid the uncontrolled activation of effector T cell responses. However, under some circumstances, such as during a rapidly dividing or systemic infection, these may become a hindrance to achieving the necessary strength and rapidity of the effector response. Thus, non-cognate stimulation of conventional T cells has been described in a number of model systems. Non-cognate interactions are defined negatively as any stimuli without TCR recognition of cognate peptide-MHC complexes presented on APCs. This type of T cell activation has often been referred to as “bystander activation” (19–22). This name seemingly refers to the idea that these are T cells which just happen to be in proximity to inflammatory stimuli that is perhaps intended for other cognate T cells, thus, assuming that non-cognate stimulation is an accidental “bystander” to the conventional response. However, it is equally possible that this “bystander” response is not incidental, but instead represents an integral functional capacity of T cells to respond and recognize inflammatory stimuli that are produced under extreme stress.

The earliest descriptions of bystander activation focus on cytokine or innate stimuli that drive T cell proliferation in the absence of antigen (20, 21, 23). These innate stimuli are also referred to as microbe-associated molecular patterns (MAMPs), and include toll-like receptor (TLR) and NOD-like receptor (NLR) ligands among others. However, it should be noted that most of this early work was completed in viral infection models, focuses largely on CD8 T cells, and does not differentiate between naïve and previously activated T cells. Thus, it is difficult to conclude from these studies whether naïve T cells can actually be primed by non-cognate interactions, particularly naïve CD4 T cells. Further, these data must be interpreted with caution, since cytokine-induced proliferation may not lead to an effector T cell state, and especially given the evidence that these same signals can induce apoptosis (20). Transient expression of the early activation marker CD69 was observed in naïve CD8 after Type I IFN stimulation, but neither this activation was not maintained nor was it shown to induce effector functional capacity (21).

In addition to non-cognate proliferation or upregulation of activation markers, there is also considerable work describing elicitation of effector functions from CD8 T cells by non-cognate stimuli, which is also confusingly referred to as bystander activation or stimulation (19, 24, 25). However, it is important to note that this work generally describes stimulation of previously activated T cells, or makes no distinction between activated and naive T cells, thus, this is not activation in the sense of initial T cell priming. For clarity, in Figure 3, a non-cognate primary activation interaction is illustrated as “bystander activation,” while a non-cognate interaction following (but separate from) a cognate primary activation interaction is referred to as “non-cognate stimulation.” This stimulation of activated T cells is discussed further below.

While most of the work described above examines CD8 T cells, less examination of TCR-independent effector responses exists specific to CD4 T cells, and most shows that bystander responses occur only in previously activated or memory CD4 T cells (26–28). There are, however, limited *in vitro* data showing that very high doses of IL-2 can make naïve CD4 T cells responsive to IL-12 or IL-18 without TCR signaling (29). Considering these distinctions, as well as the inability of many of these studies to rule out innate-like populations that were not yet identified at the time the studies were conducted, the question of whether naive T cells can be primed in a non-cognate manner under sufficient inflammatory stimulation, and whether these cells would be functional as effectors *in vivo*, lacks a definitive answer. However, non-cognate stimulation of effector and memory T cells has been shown, and some of the mechanisms are beginning to be understood.

As described in the previous section, an activated Th1 cell is primed to produce effector cytokines rapidly. Here, we focus on the effector cytokine IFN-γ, secreted by both CD8 T cells and Th1 cells. This priming means that the IFN-γ gene locus is modified to be open for faster transcription, IFN-γ mRNA has been transcribed and is ready for rapid translation, and IFN-γ protein has been translated and awaits the signals necessary for post-transcriptional modifications and secretion. Despite all of this preparation, Th1 do not constitutively secrete IFN-γ. When they receive antigen-specific TCR signals, the signaling cascade downstream of the TCR allows rapid release of the prepared IFN-γ transcripts and protein from the regulatory mechanisms that otherwise keep this production in check. However, other signaling pathways can also stimulate IFN-γ production in activated Th1.

The signals eliciting production of IFN-γ in various cell types have been studied extensively, and yet the complexity of the regulation of this cytokine continues to unfold. For example, while T-bet is considered to be a master transcriptional regulator of IFN-γ production, in natural killer (NK) cells and CD8 T cells Eomes is able to make up for the loss of T-bet, while in CD4 T cells T-bet signaling is required (30). Further, it was shown that the promoter region of IFN-γ that is utilized after TCR signaling is different than the promoter region required for IFN-γ production in response to IL-12 and IL-18 (31). In addition to transcriptional regulation, extensive post-transcriptional regulation of IFN-γ mRNA has been shown to take place, and varies between naïve, effector, and memory T cell populations (32).

While some evidence has suggested that an ability for innate ligands to interact directly with CD4 T cells to stimulate their proliferation or function (33), it is also likely that innate ligands can stimulate T cells through a second messenger that would allow amplification of the signal. Many cells are capable of responding to TLR or NLR ligands to produce inflammatory cytokines, including those cytokines known to stimulate IFN-γ production from Th1 cells. Thus, it is easy to imagine a mechanism whereby Th1 cells respond indirectly to innate stimuli by responding to inflammation. In fact, this has been shown to occur during viral infections in response to TLR ligands, and during *Salmonella* infection in response to NLR ligands, both in CD8 T cells (24, 25, 34).

Recently, we described a mechanism for innate Th1 stimulation that relies on the convergence of both TLR and NLR signaling pathways to elicit IL-18 production, which can then be recognized by activated Th1 to result in IFN-γ secretion. This mechanism is illustrated in Figure 2. Previous work has typically focused on very small populations of innately responding T cells, particularly for CD4 cells, consequently supporting the concept of the “unintended bystander.” However, the small numbers of T cells that can be seen responding at any given time during the normal course of infection do not necessarily represent a small subset of cells capable of innate response. Nor does this imply that innate stimulation of T cells occurs too infrequently to have a significant impact. Using *Salmonella* infection, we showed that Th1 cells in an infectious model of strong Th1 activation are highly susceptible to innate stimulation, with a large proportion of the Th1 capable of secreting IFN-γ in response to LPS stimulation. Finally, we demonstrated that mice whose T cells lack the capacity to be innately stimulated by IL-18 suffer a reduced capacity to clear *Salmonella* infection (35). Together, this suggests that a pathway of innate T cell response that not only can occur but must also occur for normal immune function.

### Innate-like T cells and ILCs

While the previous section focused on the non-cognate interactions of conventional αβ T cells, numerous cell types have been described that have overlapping surface markers, developmental lineages, transcription factor profiles, or effector functions to conventional T cell subsets, but which respond in a non-conventional manner (36). These cells are often thought of as innate-like cells with adaptive-like functions that can provide critical assistance in the early immune response. Examples include natural killer T (NKT) cells, mucosal-associated invariant T (MAIT) cells, γδ T cells, and innate lymphoid cells (ILCs).

Innate lymphoid cells are a rapidly expanding group of cells defined predominantly by their lack of lymphocyte antigen receptors (TCR or BCR, B cell receptor) or lineage-specific markers (36). Recently, a uniform nomenclature for ILCs was proposed that divides the various cells into three main groups. Much like the CD4 T helper subsets, Group 1 ILCs can be characterized by the expression of T-bet and IFN-γ, Group 2 express GATA3, and Group 3 express RORγt and produce IL-17 or IL-22 (37). Most ILCs require IL-7R signaling and express the surface marker CD90 and the transcription factor ID2, although it has been proposed in mice that IL-7 may inhibit transition of ILC3 to ILC1 (38). Further elaboration will center on the Group 1 ILC subset, given its similarity to the Th1 subset of CD4 T cells.

Natural killer cells have been included within this nomenclature as a Group 1 ILC, alongside ILC1. While NK cells have been very well-described as an early source of IFN-γ and TNFα, in addition to their cytotoxic functions, very little is known about ILC1. Although there is evidence that they develop from ILC3s after IL-12 stimulation, it was recently shown that ILC1 can also develop independently from a common innate lymphoid progenitor (39). However, whether they arise separately or as a consequence of functional plasticity, it remains that there is a group of T-bet+ ILCs, which can respond to IL-12 and IL-18 signals to produce IFN-γ, but which are not NK cells and do not exhibit cytotoxicity (37, 38). As a whole, the early innate effector responses of the Group 1 ILCs during intracellular infections play a key role in host protection, inflammation, and initiation of adaptive responses.

While ILCs lack a TCR, several other cell types express unique TCRs that allow for a non-conventional response, known collectively as “innate-like T cells” due to their ability to respond rapidly to innate stimulation. Among these innate-like T cells, one common method allowing for a non-cognate-antigen response is an invariant or semi-invariant TCR. While conventional TCRs undergo recombination activating gene (RAG) dependent rearrangement of their α and β chains during development in the thymus to allow for a broader repertoire with improved specificity, some innate-like cell populations possess TCRs with single α-chain and restricted β-chain specificities. Particular examples of cells with these alternative TCRs include invariant NKT cells (iNKT) and MAIT cells (36).

Natural killer T cells are perhaps the best described of these innate-like T cell subsets. NKT cells are innate-like T cells in the sense that they develop in the thymus and express a TCR, but they also express NK1.1 and several innate activating or inhibitory receptors typically found on NK cells (40). Two types of NKT cells exist: NKT I are the well-described, iNKT cells known to respond to lipids and especially with high affinity to α-galactosylceramide presented by the MHC-related molecule CD1d, while NKT II are less studied, to date, but have a diverse TCR repertoire and fail to respond to α-galactosylceramide (41). While iNKT can be activated by CD1d presentation of foreign lipid antigens, they may also be activated by CD1d presentation of lipid self-antigens and require inflammatory cytokine signals, allowing for more rapid and innate-like responses (40–42). Further, it was recently shown that iNKT can be activated in an antigen-independent manner by cytokine alone during some infections, like *Salmonella*, but not others (43).

Another semi-invariant T cell population are the recently described MAIT cells, characterized by their localization to mucosal tissue and their recognition of the MHC-related molecule, MR1, which binds the metabolites of B vitamins generated by bacteria and fungi (36). MAIT cells develop and are pre-programed in the thymus, but quickly acquire an activated phenotype in the periphery. There is now evidence to suggest that this activation occurs in response to microbiota; in particular, the observation that germ-free mice have diminished numbers of MAIT cells, which can be recovered upon monoculture reconstitution with many bacteria or yeast, but not *Enterococcus faecalis*, which lacks the riboflavin metabolic pathway. Unlike NKT, they seem to respond predominantly to TCR ligation and do not require cytokine stimulation to elicit effector functions, which consist mostly of IFN-γ and TNFα, although they can also express IL-17 (44, 45).

γδ T cells are a unique exception, in that they possess recombined TCRs, but can respond in a rapid, innate-like manner to inflammatory cytokines. Thus, these T cells are technically a component of the adaptive immune response, but are often discussed in the context of early innate responses (46). Differentiation programing of γδ T cells occurs during thymic development, determining either an IFN-γ, IL-17, or IL-4 producing phenotype (47), but peripheral activation is still required before effector functions can be elicited. The relative contributions of TCR, costimulation and cytokine signals to this activation still seem to be a matter of some debate, and may be partially dependent on the subset, but whatever the mechanism these cells respond far more rapidly than their αβ T cell counterparts (46).

All of these innate-like T cells (iNKT, MAIT, and γδT), although possessing different TCRs and recognizing different antigen repertoires, share some common features. For one thing, in each cell type the ability to generate or maintain immunological memory is poorly defined, as are the required signals for survival and proliferation (36). For both iNKT and γδ T cells functional subsets have now been described analogous to the CD4 T helper subsets (40, 47), although unlike CD4 T cells these subsets are pre-determined during development in the thymus. Although subsets have not yet been defined as such for MAIT cells, and they typically respond to IL-12 to produce IFN-γ in a T-bet-dependent manner, they also express RORγt and can express IL-17 and IL-22 under appropriate stimulation. Further, there is now some evidence for an immunoregulatory function of MAIT cells. Thus, whether MAIT cells have functional subsets or are simply functionally promiscuous remains to be determined (44, 45).

In further similarity, each is described as “innate-like” due to an ability to rapidly respond to innate stimulation – that is, they respond to the inflammatory cytokines that result from innate stimuli. However, for each cell subset the specific requirements of initial priming, and in particular whether this priming can occur without any peripheral TCR stimulation, is still a matter of debate within their respective fields. While earlier literature suggested that these cells respond rapidly because they are able to respond to cytokine alone, other work shows that these cells require TCR interactions (48), and for iNKT at least this requirement can be met by self-antigens under inflammatory conditions to allow more rapid responses (40–42). More recent evidence suggests that these requirements may differ under varying circumstances (43). Herein, we make an argument that conventional αβ T cells can also respond rapidly to inflammatory cytokines in an innate-like manner once they have been primed. The parallels between these responses and their mechanisms suggest that a conservation of these stimulatory mechanisms between conventional and innate-like T cells, and highlight the need for a better understanding of the activation requirements of non-conventional T cells.

## A Complicated Relationship: The Dynamic Interactions of *Salmonella* and Th1 Cells

### *Salmonella*: A persistent global challenge

While many bacteria live and replicate extracellularly, entering the host cell only when engulfed and destroyed by phagocytes, some bacteria have adapted unique survival strategies to allow a protected life cycle within host cells. Some of these bacteria are obligate intracellular pathogens, like *Chlamydia*, that cannot replicate outside of the cell, but many intracellular bacteria are capable of occupying either space. The immune system has, in turn, developed a number of ways to recognize pathogens within cells, pathways which the pathogen actively attempts to thwart (49).

In this review, we focus on *Salmonella*, a Gram-negative enteric pathogen that resides predominantly within the phagosomes of macrophages located in the spleen, liver, and bone marrow. In human beings, there are two forms of systemic salmonelloses: typhoid fever and non-typhoidal salmonellosis, or NTS. Typhoid and paratyphoid fevers are caused by the human-specific pathogens *Salmonella enterica* serovar Typhi and Paratyphi, still occur endemically in developing countries, and can cause severe systemic disease even in healthy individuals. Estimates range as high as 27 million annual infections with either Typhi or the clinically indistinguishable Paratyphi. In contrast, NTS occurs only in immunocompromised individuals, but can originate from any of the >2000 *Salmonella* serovars capable of causing foodborne illness in human beings and harbored in a wide variety of animal reservoirs (50). Thus, both systemic infections remain a source of concern for public health officials worldwide.

Although antibiotics effective against *Salmonella* are available, the options are relatively limited for intracellular pathogens as compared to more accessible extracellular pathogens. Additionally, among those antibiotics currently available, there is a growing incidence of drug resistance, including multi-drug resistance to the first-line treatments, and resistance to the now standard fluoroquinolones. Further, decreased susceptibility to the fluoroquinolone ciprofloxacin has been associated in enteric fever patients with prolonged fever and increased rates of treatment failure (51). Finally, an analysis of the *Salmonella* metabolic pathways has suggested that most of the major or non-redundant pathways have already been targeted or considered for drug inhibition, suggesting that a limitation to prospective future development of new antibiotic treatments (52). Together, these studies emphasize the need for alternative treatment options and for improved vaccination strategies that could lessen the need for, and consequently the selective pressure upon, traditional antibiotic therapy.

Currently, two vaccinations are commercially available in the U.S. for travelers to typhoid endemic countries. One is a Vi capsular polysaccharide (ViCPS) vaccine administered intramuscularly as one dose at least 1 week prior to exposure. The second is an oral, attenuated Ty21a vaccine available under several formulations, typically administered every other day as three separate doses 2 weeks prior to exposure. Both vaccinations suffer from limitations that impair their practicality in typhoid endemic regions, not the least of which is the need for regular re-vaccination, and the low-reported efficacy at 3 years of 51–55% (53). The ViCPS vaccine is approved in children over the age of 2 years old, and the oral vaccine for children over the age of 5 years, while repeated exposure before the age of 5 in endemic areas has been shown. This suggests that the vaccines available miss a key population.

In addition, while evidence suggests that Ty21a may be cross-protective for paratyphoid, the ViCPS vaccine targets an antigen that does not exist in Paratyphi and even some strains of Typhi (51, 54). Further, because the oral vaccine is a live, attenuated *Salmonella* strain, it is not suitable for use in immune-compromised patients, posing a challenge to widespread use in areas co-endemic for HIV. Thus, currently available vaccination strategies are not adequate to allow control of systemic typhoidal disease (53). Whether currently available vaccines mediate any protection to non-typhoidal systemic diseases has not been thoroughly characterized. These data emphasize a need to better understand the immune response during systemic *Salmonella* infections, to inform better vaccine design.

### Importance of Th1 cells and IFN-γ in intracellular infections

As mentioned earlier, some bacteria and other pathogens have developed the capacity to reside within cells and effectively hide from extracellular immune recognition. Often, these pathogens enter the cells initially using the cells’ own phagocytic capacity, but then are able to escape phagolysosomal degradation or escape the phagosome entirely, by a wide range of different mechanisms (49, 55). Given this unique lifestyle, intracellular pathogens require a special type of immune response designed to recognize infected phagocytes and mediate either killing of the infected cell or internal pathogen killing mechanisms. CD8 T cells have cell-specific cytotoxic capacity, allowing directed killing of infected cells, while both CD8 and CD4 T cells can secrete pro-inflammatory cytokines that activate phagocytes to initiate internal mechanisms of pathogen destruction.

CD8 T cells have multiple cytolytic capacities initiated by TCR interactions, including release of secretory granules and death receptor-mediated apoptosis. However, while these responses have a critical role in anti-viral defenses, their role against other intracellular pathogens is limited (56). Of more importance to intracellular bacterial infections, CD8 can produce pro-inflammatory cytokines such as IFN-γ, which activates macrophages to undergo changes that alter the intracellular environment to become less hospitable to the invading pathogen. While CD8 T cells respond to MHC-I complexes that mostly present antigen processed from the cytosol of nearly all cell types, CD4 T cells respond to MHC-II presented antigen on special APCs derived from the endocytic pathway. This allows CD4 T cells to recognize antigens from pathogens hiding inside of cells within endosomes, as well as antigens taken up from outside of the cell (57). Reliance on different antigen processing pathways and consequent MHC presentation allows a partial division of labor between the CD8 and CD4 T cells, although they have retained some redundancy in critical functions, such as production of IFN-γ.

Among the various CD4 T cell subsets, the CD4 Th1 cells provide the primary response to intracellular pathogens. As mentioned earlier, after CD4 T cells are activated they receive a differentiation signal that determines their cytokine profile. In Th1 cells, IL-12 upregulates the transcription factor T-bet, which is required by CD4 T cells for IFN-γ production (58). Once activated, Th1 cells are programed to secrete pro-inflammatory cytokines that include IFN-γ, TNFα, and IL-2 upon re-stimulation. In contrast, CD8 T cell priming is not usually thought of in terms of differentiation of particular subsets with distinct functions, although functional subsets have been described. Termed Tc1, Tc2, and Tc17 in reference to their Th counterparts, these cells are found in relatively low-frequency under normal circumstances, and the mechanisms driving the development of these alternative CD8 T cells remain poorly understood (58). Signal 3 was initially identified in CD4 and CD8 T cells simply as the inflammatory cytokine(s) required to induce proliferation and differentiation to effector capacity (59). Type I interferons and IL-12 have both extensively been shown to result in the survival, expansion, and differentiation of CD8 T cells, and are critical for the conventionally described CD8 T cell effector responses, including cytolytic activity and IFN-γ production (59–62).

Additional differences between CD4 and CD8 T cell activation include, but are not limited to a shorter required duration of antigenic stimulation in CD8 T cells (63–65), different transcriptional regulation, including partial redundancy for the transcription factors T-bet and Eomes in IFN-γ production in CD8, but not CD4 (30), and differences in cellular trafficking and antigen surveillance (66). Further, evidence suggests that CD4 T cells can help to initiate CD8 T cell priming (67, 68), and may be required for optimal CD8 memory formation, a regulatory interaction that argues against mechanistic redundancy in the activation process. Thus, while T cells have evolved to share many similar pathways, CD4 and CD8 T cells are distinct cell types with different functions and rules to govern them. When studying T cell functions it is critical to keep these differences in mind and to choose a model system capable of demonstrating the full potential of the cell of interest. Accordingly, the strong requirement for Th1 cell functions in *Salmonella* infections makes this model system ideal for study of Th1 responses (9–11).

The cytokine IFN-γ is especially important during intracellular infection because of its critical capacity to activate macrophages to become M1, or classically activated, macrophages. M1 macrophages modify their internal environment to become as inhospitable as possible, including production of anti-microbial compounds like reactive oxygen and nitrogen species, as well as themselves secreting pro-inflammatory cytokines (69). The importance of IFN-γ-mediated macrophage activation is highlighted by the effects, resulting from the loss of IFN-γ or IFN-γ-inducing cytokines and transcription factors (11, 70, 71). In both mice and human beings, loss of IFN-γ results in an inability to effectively clear intracellular pathogens (72). Further, in human beings with chronic granulomatous disease or mycobacterial granulomas, IFN-γ is an effective, albeit toxic, therapeutic (73–75). Combined, these studies clearly demonstrate the requirement of IFN-γ for effective clearance of intracellular pathogens.

### Playing hard to get: How *Salmonella* subverts the Th1 cell response

**Figure 4:**
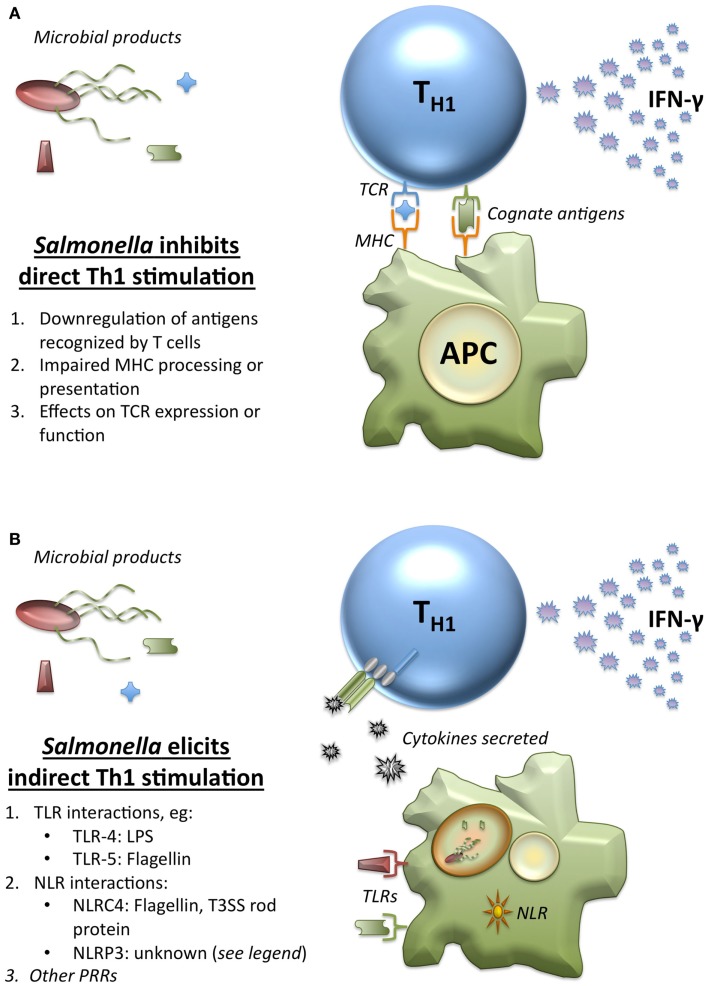
**Direct and indirect stimulation of Th1 by *Salmonella***. **(A)** *Salmonella* has developed multiple mechanisms to inhibit direct Th1 stimulation by cognate antigen recognition. Conventionally, *Salmonella*-specific antigens would be processed and presented by APCs on MHC-II to the TCR of Th1 cells to elicit IFN-γ at sites of infection. However, *Salmonella* has developed a wide array of strategies to limit this interaction. This includes (1) downregulation of antigens that were expressed upon entry into the host that T cells may have been primed to recognize, (2) mechanisms designed to impair processing and presentation of antigen by APCs, including downregulation of MHC itself, and (3) effects upon the expression or function of the TCR. These strategies aim to block cognate antigen recognition each step of the way, making additional non-cognate mechanisms for T cell stimulation crucial. **(B)** Many innate immune recognition pathways respond to *Salmonella*, and these elicit cytokines that allow Th1 cells to be stimulated by non-cognate pathogen products indirectly at sites of infection. (1) Several TLRs recognize conserved patterns present in *Salmonella*, including TLR-4 recognition of LPS, TLR-5 recognition of flagellin, and other TLRs that recognize bacterial nucleic acids from within the endolysosome. (2) The role of NLRs and the inflammasomes have also been well-demonstrated for *Salmonella*. While NLRC4 is known to recognize both flagellin and T3SS (Type 3 secretion system) rod proteins, the exact ligand recognized by NLRP3 from *Salmonella* is unknown. NLRP3 recognizes a number of nuclear components, which act as danger signals when present in the cytosol, but other ligands have been proposed. (3) Although less well-characterized, other classes of PRRs have the potential to recognize *Salmonella*, including the various cytosolic nucleic acid sensing receptors.

The critical role of Th1 cells in *Salmonella* clearance makes them an obvious target for immune evasion strategies. While there is extensive information available on the ways that *Salmonella* has found to manipulate the system (76, 77), we focus here particularly on immune evasion strategies that impair the ability for T cells to recognize their specific antigen. Three of the ways that *Salmonella* have developed to achieve this include downregulation of antigens that may be recognized by T cells, effects on TCR expression or function, and impairment of MHC processing or presentation of peptides (Figure 4A). The active avoidance of TCR recognition employed by *Salmonella* provides one possible explanation for the maintenance of a TCR-independent T cell stimulatory pathway.

When *Salmonella* change environments, from contaminated source to intestine to myeloid cells, their expression of antigens recognized by T cells rapidly changes (8). In addition to responding to a change in needs, this antigenic shift acts as a highly effective immune evasion strategy, resulting in the activation and expansion of large numbers of T cells that specifically recognize antigens that will not be present at the site of infection. The T cell response to FliC was shown to be inefficient at resolving infection over a decade ago, and both SipC and FliC elicit an early T cell response despite rapid downregulation of these antigens by *Salmonella* (7, 78, 79). Thus, much of the early T cell response may develop to antigens that are not available at the site of infection, preventing cognate activation for these cells.

Additionally, a number of mechanisms have been demonstrated by which *Salmonella* causes downregulation of the TCR on T cells (80). Flagellin stimulation has been shown to result in upregulation of SOCS1, which impairs TCR expression in T cells (81). Further, direct contact of *Salmonella* with T cells results in secretion of the enzyme l-asparaginase II by *Salmonella*, which breaks down l-asparagine and consequently impairs T cell blastogenesis, proliferation, and cytokine secretion by downregulating the TCR β chain (82, 83). While these mechanisms may impair the initial priming of T cells, the requirement for direct contact between T cells and bacteria in some of these studies suggests that the importance of these evasion techniques at sites of infection.

Finally, many strategies have been demonstrated by which *Salmonella* is able to inhibit either the processing of antigens into peptides or the presentation of these antigenic peptides on the surface of APCs within the MHCs (80, 84). Nearly 20 years ago, the two-component regulatory system member PhoP was shown to impair processing and presentation of antigens in macrophages (85). Further, numerous *Salmonella* pathogenicity island-2 (SPI-2) effector proteins have been implicated in impaired MHC function, including impaired loading of peptides onto MHC, prevention of lysosomal degradation that results in decreased peptide availability, and polyubiquitination of MHC that results in degradation rather than surface expression (86–89). Each of these interactions targets a step in the antigen presentation pathway that ultimately results in an impaired ability for infected cells to signal to T cells.

Given the hindrance of cognate T cell stimulation, the ability for T cells to be stimulated by non-cognate interactions as well could play an important role in *Salmonella* clearance. *Salmonella* induces a number of different non-cognate responses via PRRs, as outlined in Figure 4B. These include well-characterized TLR and NLR interactions, as well as other, less well-defined PRR interactions (90). Recognition of these various non-cognate ligands results in inflammation, including production of inflammatory cytokines that can stimulate T cells. This indirect stimulation of T cells in response to non-cognate *Salmonella* products provides a complementary mechanism for T cell stimulation at sites of infection with a broad array of conserved triggers. While the multitude of Th1 evasive mechanisms accentuates the need for innate signaling pathways in the elicitation of Th1 cell functions, it is important to note that innate immune pathways are not exempt from evasion strategies (91, 92). Thus, in order to provide T cells the best chance to encounter and respond to signs of infection, redundant mechanisms for stimulation that rely on either cognate antigen or MAMP-driven inflammation have developed.

## Discussion

Herein, we have discussed a number of advantages to studying Th1 cell responses in a *Salmonella* model. The strong understanding of both CD4 T cell and innate immune pathways during the response to *Salmonella* infection provides a strong foundation to further explore the potential interaction between these pathways. Moreover, the tools for these studies are readily available in such a well-studied and easily manipulated model pathogen. Together, these factors make *Salmonella* an ideal model pathogen for the study of non-cognate Th1 cell responses.

Furthermore, we highlight a number of questions that remain to be answered in the study of non-cognate T cell interactions. One such question: what is the fate of Th1 cells after non-cognate stimulation? While it is possible that cells undergo the same response and fate following either cognate or non-cognate secondary interactions, there is still limited understanding of the effect of multiple stimulatory interactions with T cells. It is possible that the heightened effector response that occurs after re-stimulation impacts the fate decisions of effector T cells. It is also possible that the combination of cognate activation and non-cognate stimulation could regulate cell fate differently than solely cognate interactions. If that is the case, non-cognate stimulation could result in apoptosis and the subsequent loss of these cells, or it could act as a signal for a cell that should transition to memory during contraction. Within the memory pool, there may be a subtle difference between cells that did or did not receive non-cognate stimulation during their effector phase. Further work is required to determine what the impact of this response pathway is on T cell fate decisions.

Additionally, more work is necessary to clarify the requirements and role of non-cognate interactions of memory CD4 T cells. Our data have shown that the response of memory T cells to LPS alone after *Salmonella* clearance is much lower than the response observed during infection (35). However, it is not yet clear why so few memory T cells were able to respond. Could they require cognate re-activation first, and if so, why? Does the active infection contribute something necessary for non-cognate T cell stimulation, such as inflammasome activation or an additional cytokine? If something is missing, why are any memory T cells able to respond to the stimulation? Is there something different about these memory cells that retain innate stimulatory capacity? Understanding the memory response is critical to understanding the role of non-cognate stimulation, in particular, when trying to apply these findings to the improvement of vaccine strategies or to understanding a possible role in autoimmune disease.

Finally, while much of the early work in describing cognate T cell interactions was done *in vitro*, recent advances in the capacity of live *in vivo* imaging technologies have allowed for real-time observations of these complex interactions. This system is advantageous because it allows for individual cell tracking and a chronological history of a specific cell under natural conditions. For example, during the activation process an individual T cell may interact with many separate DCs, and these interactions have a cumulative effect upon T cell function (63). Further, it allows exploration of such brief conversations as occur, for instance, at the kinapse, which are otherwise difficult to capture due to their transience (93). Unfortunately, there are currently technical limitations that could make visualizing T cell responses to *Salmonella* difficult. As the technology continues to improve, studies such as these will open the door for a new understanding of the dynamic complexity of T cell interactions within the contexts of time and space that have, until now, proven particularly challenging for immunologists (94). Similarly, it may soon be possible to watch cognate and non-cognate T cell interactions as they occur to begin to answer questions about non-cognate Th1 stimulation such as those posed here.

As the mechanisms underlying non-cognate Th1 stimulation become clearer it may also become possible to define the relative contributions of cognate and non-cognate T cell interactions at various stages in the Th1 response. For example, in Section “Bystander Activation and Non-Cognate Stimulation,” we discuss recent evidence that Th1 cells require IL-18 receptor signaling in order to respond to non-cognate signals but not for initial TCR-dependent activation. These differential pathways provide one possible tool for separating cognate and non-cognate response roles. To deeply explore these responses and their outcomes, a system in which each pathway could be selectively and transiently inhibited would be ideal. Such studies could be critical to understanding the role of infection and inflammation in T cell responses.

## Conflict of Interest Statement

The authors declare that the research was conducted in the absence of any commercial or financial relationships that could be construed as a potential conflict of interest.
